# Identification of maturation and protein synthesis related proteins from porcine oocytes during in vitro maturation

**DOI:** 10.1186/1477-5956-9-28

**Published:** 2011-06-08

**Authors:** Jumi Kim, Ji-Su Kim, Young-Joo Jeon, Dong-Wook Kim, Tae-Ho Yang, Yunjo Soh, Hak Kyo Lee, Nag-Jin Choi, Soo-Bong Park, Kang Seok Seo, Hyung Min Chung, Dong-Seok Lee, Jung-Il Chae

**Affiliations:** 1 CHA Bio & Diostech Co., Ltd. 606-16 Yeoksam 1 dong, Gangnam gu, Seoul 135-907, Korea; 2 National Primate Research Center, Korea Research Institute of Bioscience and Biotechnology, Ochang, Chungbuk 363-883, Korea; 3 Department of Dental Pharmacology, School of Dentistry, Brain Korea 21 Project, Chonbuk National University, Jeonju, 561-756, Korea; 4 Division of Biological Sciences, Chonbuk National University, Jeonju 561-756, Korea; 5 Genomic Informatics Center, Hankyong National University, 67 Sukjong-dong, Ansung-city, Kyongi-do, 456-749, Korea; 6 Department of Animal Science, College of Agricultural & Life Science, Chonbuk National University, Jeonju, Korea; 7 National Institute of animal Science, Suwon 441-706 Korea; 8 Department of Animal Science and Technology, Sunchon National University, Suncheon 540-742, Korea; 9 Graduate School of Life Science, CHA Stem Cell Institute, College of Medicine, CHA University, 605 -21 Yeoksam 1 dong, Gangnam gu, Seoul 135-907, Korea; 10 College of Natural Sciences, Kyungpook National University, Daegu 702-701, Korea

**Keywords:** Porcine Oocytes, In Vitro Maturation, proteomics

## Abstract

**Background:**

In vitro maturation (IVM) of mammalian oocytes is divided into the GV (germinal vesicle stage), MI (metaphase I stage) and MII (metaphase II stage) stages, and only fully mature oocytes have acquired the ability to be fertilized and initiate zygotic development. These observations have been mostly based on morphological evaluations, but the molecular events governing these processes are not fully understood.

The aim of the present study was to better understand the processes involved in the molecular regulation of IVM using 2-DE analysis followed by mass spectrometry to identify proteins that are differentially expressed during oocyte IVM.

**Result:**

A total of 16 up-regulated and 12 down-regulated proteins were identified. To investigate the IVM process, we specifically focused on the proteins that were up-regulated during the MII stage when compared with the GV stage, which included PRDX 2, GST, SPSY, myomegalin, PED4D, PRKAB 1, and DTNA. These up-regulated proteins were functionally involved in redox regulation and the cAMP-dependent pathway, which are essential for the intracellular signaling involved in oocyte maturation. Interestingly, the PDE4D and its partner, myomegalin, during the MII stage was consistently confirmed up-regulation by western blot analyses.

**Conclusion:**

These results could be used to better understand some aspects of the molecular mechanisms underlying porcine oocyte maturation. This study identified some regulatory proteins that may have important roles in the molecular events involved in porcine oocyte maturation, particularly with respect to the regulation of oocyte meiotic resumption, MII arrest and oocyte activation. In addition, this study may have beneficial applications not only to basic science with respect to the improvement of oocyte culture conditions but also to mammalian reproductive biotechnology with potential implications.

## Background

The pig is not only an important farm animal but also an animal model used in biomedical research including reproductive technology. In recent years, pig oocytes have been extensively used in basic research on reproductive techniques, oocyte development and the generation of cloned or genetically modified animals because of their high availability and unique characteristics[1]. Occasionally, these studies fail or have a low experimental efficiency due to problems with oocyte in vitro maturation (IVM) that result from incomplete cytoplasmic maturation. IVM is characterized by an initial breakdown of the germinal vesicle (GV) and the rearrangement of microtubule networks during the first meiosis (MI), followed by extrusion of the first polar body and subsequent arrest of the oocytes in metaphase during the second meiosis (MII)[2,3]. To improve the efficacy of oocyte IVM, several systems have been established to define the in vitro conditions for oocyte maturation and fertilization. These efforts, however, have not identified optimal conditions for oocyte IVM, thus limiting their application to other reproductive techniques in pigs. Thus, defined *in vitro *conditions for oocyte maturation should be identified and developed. Two important aspects involved in characterizing the conditions for IVM are discovering key regulatory factors and understanding the molecular mechanisms involved in oocyte maturation. Unfortunately, few studies have focused on these aspects, and thus little is known about the underlying molecular networks and mechanisms of oocyte maturation. Approaches such as genomics and proteomics may provide a better understanding of oocyte maturation that can be applied to improve the poor developmental potential of oocytes produced *in vitro*, leading to their successful maturation and fertilization [4,5].

There were relatively few studies that have examined the genomes and proteomes of germ cells, embryos and whole tissues important for reproductive function due to the limited availability of sample cells and lack of sufficiently sensitive procedures. Several technological advances now allow these genomic and proteomic analyses to be used to study a wide variety of biological samples, including those samples that are extremely limited [6].

Cellular maturation and differentiation processes are characterized by the expression of specific proteins. However, there may not be a consistent linear correlation between mRNA and protein levels, particularly in oocytes. Furthermore, protein activity can be affected by post-translational modifications, such as those regulated by specific kinases and phosphatases. Post-translationally modified proteins are involved in many cellular processes, including cell growth, differentiation, the cell cycle and meiosis. The activation of certain protein kinases, such as maturation promoting factor (MPF) and mitogen-activated protein kinase (MAPK), plays a significant role during the meiotic maturation of oocytes.

The activation of certain protein kinases, such as cyclin dependent kinase 1 (Cdk 1) and mitogen-activated protein kinase (MAPK), plays a significant role during the meiotic maturation of oocytes.

The activation of maturation promoting factor (MPF) and mitogen-activated protein kinase (MAPK), including cyclin dependent kinase 1 (Cdk 1), extracellular signal regulated kinases 1(ERK 1) and ERK 2, plays a significant role during the meiotic maturation of oocytes. This proteomic approach will be used to investigate protein expression changes during oocyte maturation that could be used to identify key regulatory proteins that are predominantly expressed during the oocyte maturation process, which in turn may contribute to improvements in oocyte IVM efficacy [7-9]. The molecular events responsible for these processes, however, are still poorly unknown.

The aim of the present study was to identify regulatory proteins involved in oocytes IVM using proteomic analyses. This approach will be used valuably to better understand the IVM process and to improve IVM efficacy.

## Results

### In Vitro Maturation of Porcine Oocytes

**Figure 1:**
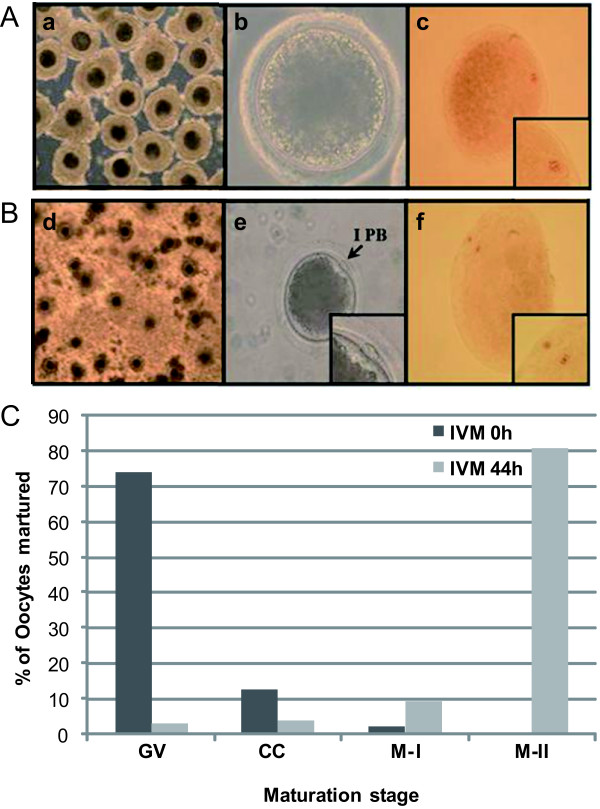
**Morphological characterization and orcein staining of porcine oocytes during IVM**. **(**A) Oocyte released from a follicle containing an intact germinal vesicle (GV). (B) The M II stage and extrusion of the first polar body (I PB). Morphological characterization of the porcine oocytes in each stage (a, b, d, and e), and morphological evaluation of each of these stages using orcein staining (c, f). (C) Changes in the nuclear status of oocytes cultured in TCM-199 medium throughout the process of IVM. The oocytes were examined at four different stages of maturation: germinal vesicle (GV), chromosome condense (CC), metaphase I (M I) and metaphase II (M II).

To investigate protein expression changes during IVM, pig oocytes were cultured in vitro to induce oocytes maturation. The different stages involved in oocytes IVM are referred to as the GV, MI and MII stages, which were distinguished by morphological analyses. Oocytes, which were cultured over a 44-hr period, were divided into GV- and MII-stage oocytes also using morphological analyses. Under the TCM-199-based culture system supplemented with the hormones hCG, EGF, LH and FSH, the porcine oocytes released from their follicles at the GV stage at 24 h, which initiated GV breakdown (GVBD), with the majority of oocytes reaching the M I stage by 28 h (data not shown). Following this phase, the oocytes progressed through the anaphase I/telophase I stages, and by 44 h, almost all the oocytes reached the M II stage, with the typical extruded first polar body (IPB) generally present (Figure 1A, B). At the initiation of maturation, 74.3% and 2.1% of the oocytes were in the GV and M II stages, respectively. After the onset of maturation by hour 44, 81% of oocytes were in the M II stage (Figure 1C).

Based on these morphological observations, homogenous cell populations of oocytes in either the GV or MII stages of IVM were collected. These two populations were used to prepare samples for 2-DE analyses in duplicate.

### 2-DE Proteomic Analysis of Porcine Oocytes

**Figure 2:**
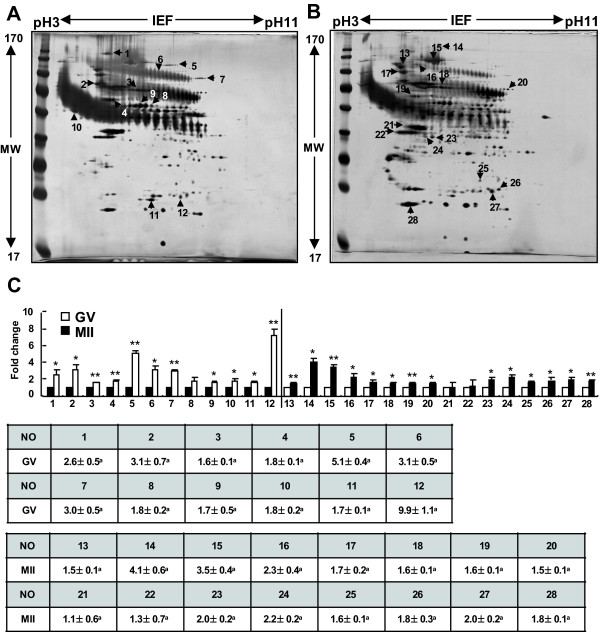
**Two-dimensional protein gels of GV- and M II-stage porcine oocytes**. The proteins were separated using 2-DE gel electrophoresis, with the first dimension involving an 18-cm pH 3-11 NL IPG and the second dimension involving 10% gels (A: GV; B: MII), and visualized by silver staining. (C) Comparison of spot intensity in the 2-DE gels for the GV and MII-stage oocytes using quantitative analysis. The results, which were analyzed by the Phoretix Expression software, are presented in a bar graph *Value significantly differs from the control (*P < 0.05 and **P < 0.01). The numbered spots were identified by mass spectrometry, and the numbers correspond to their respective proteins in Tables 1 and 2.

2-DE protein separation coupled with protein identification by mass spectrometry was used as a classic proteomic approach to investigate protein expression changes during the GV and MII stages of porcine oocyte IVM. Over 1200 GV- and M II-stage oocytes each were used in this analysis. Total protein extracts were separated on 18-cm, nonlinear pH 3-11 IPG strips and 10% SDS-PAGE, which resolved proteins over a 17-kDa mass range. In this phase of the study, more than 250 protein spots from the GV- and MII-stage oocytes were detected and mapped on each silver-stained gel. Representative 2-DE images are shown in Figure 2.

A total of 50 protein spots showed at least a 1.5-fold expression difference between the GV and MII stages. These differentially expressed protein spots were selected for further protein identification using mass spectrometry. As an additional criterion for inclusion in the mass spectrometry analysis, only those protein spots confirmed in duplicate on different silver-stained gels were considered for protein identification.

### Identification of Oocyte Proteins by MALDI-TOF/MS and LC MS/MS

The differentially expressed spots were excised from the gels and identified after tryptic digestion using MALDI-TOF/MS and LC MS/MS, and candidate proteins matching the tryptic peptide masses were identified using the Mascot program. In most cases, the experimental isoelectric points (pI) and molecular weights (MW) of the proteins were in agreement with their theoretical values, as determined using MSDB and NCBI databases. Some of the spots contained incomplete polypeptide fragments and could not be identified.

**Figure 3:**
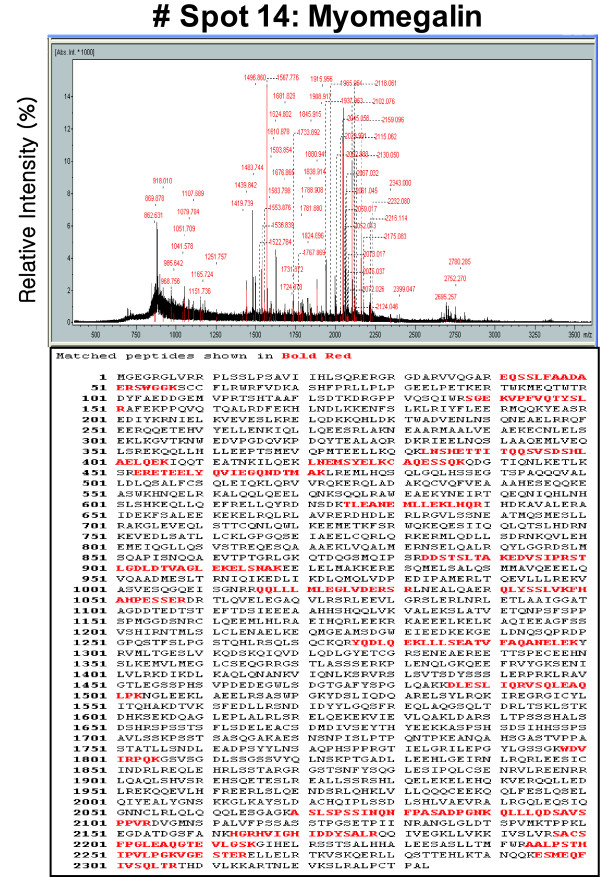
**MALDI-TOF MS peptide mass fingerprint spectrum of spot No. 14 (gi|114558492)**. The protein corresponding to spot No. 14 in Figure 2 was extracted from the 2-D gel and analyzed by MALDI-TOF MS to determine its identity.

The accuracy of our system was in the range of ± 0.05 Da. For peptide sequencing, the number of peptides analyzed and the Mascot score are specified. A representative fragment spectrum from the MALDI-TOF MS analysis is shown in Figure 3. Only results that were statistically significant at the 5% level were considered (p < 0.05).

A total of 28 proteins were identified from the 50 differentially expressed protein spots. In the stage MII porcine oocytes during IVM, 16 spots were present at higher levels (1.65- to 5.28-fold increases), and 12 spots were present at lower levels (1.75- to 7.56-fold decreases or not detectable) when compared with the control GV stage oocytes. These results suggested that the porcine IVM process depends upon 28 specific proteins that are differentially expressed. These proteins may have important roles in the molecular events involved in porcine oocyte development.

The down-regulated proteins included the following: Heat shock 70 kDa protein(HSP 70), glucose-regulated protein precursor (GRP 78) isoform 1, TD and POZ domain-containing protein 1, protein disulfide isomerase, M-phase phosphoprotein 1 (MPP1), Chain A, Steric And Conformational Features Of The Aconitase Mechanism, Zona pellucida sperm-binding protein 3 precursor, zona pellucida glycoprotein 4, Cerebellar degeneration-related protein 2, peroxiredoxin 3 and heat shock protein 27 kDa (HSP 27) (Table 1).

**Table 1:** Proteins down-regulated in the M II stage during IVM.

**Spot No**.	Protein name	NCBI Accession **No**.	Swiss-prot Accession **No**.	Type of analysis	PMF (MS)		Theoretical		P-value
						
					Score	% Coverage	MW [Da]	pI	
1	similar to G patch domain and KOW motifs, partial	gi|114688528		MALDI-TOF	74	45	23,984	6.54	0.031
2	Heat shock 70 kDa protein 5 glucose-regulated protein precursor (GRP 78) isoform 1	gi|114626692		MALDI-TOF/TOF	123	32	68,354	5.15	0.031
3	TD and POZ domain-containing protein 1	gi|57013061		MALDI-TOF	64	29	99,551	5.34	0.003
4	protein disulfide isomerase Protein disulfide-isomerase A3 precursor (ERP57)	gi|860986		MALDI-TOF	84	24	57,043	6.1	0.008
5	M-phase phosphoprotein 1 (MPP1)	gi|124015169	Q80WE4	MALDI-TOF	51	13	204,969	5.61	0.003
6	unnamed protein product	gi|90076858		MALDI-TOF	98	32	34,313	6.04	0.017
7	Chain A, Steric And Conformational Features Of The Aconitase Mechanism	gi|157829964		MALDI-TOF	87	26	86,449	8.24	0.003
8	Zona pellucida sperm-binding protein 3 precursor (Zona pellucida glycoprotein ZP3, Sperm receptor)	gi|1353191	P42098	LC-MSMS	51	7	44,959	6.17	0.127
9	zona pellucida glycoprotein 4	gi|47522906	Q07287	LC-MSMS	65	3	59296	8.47	0.011
10	Cerebellar degeneration-related protein 2	gi|67460136		LC-MSMS	155	12	52068	4.94	0.027
11	peroxiredoxin 3	gi|27806083	Q9BGI3	LC-MSMS	204	21	28,117	7.15	0.01
12	heat shock protein 27 kDa	gi|50916342	Q5IZV0	LC-MSMS	75	20	14,211	5.94	0.004

The up-regulated proteins included the following: protein kinase 5'-AMP-activated protein kinase subunit beta-1, Myomegalin, major vault protein, Heat shock protein HSP 90-alpha 2(HSP 90-α), heat-shock protein hsp86, heat shock protein 70.2, phosphoglucomutase 5, Dystrobrevin alpha (DTNA), cytoskeletal beta actin, spermine synthase (SPSY), galactokinase I, transferase, HG -phosphoribosyl, glutathione S-transferase, mu 2, glutathione-S-transferase, mu 5 and Peroxiredoxin-2 (Table 2).

**Table 2:** Proteins up-regulated in the M II stage during IVM.

**Spot No**.	Protein name	NCBI Accession **No**.	Swiss-prot Accession **No**.	Type of analysis	PMF (MS)		Theoretical		P-value
						
					Score	% Coverage	MW [Da]	pI	
13	protein kinase 5'-AMP-activated protein kinase subunit beta-1 [Fragment]	gi|984249	P80387	MALDI-TOF/TOF	107	25	92,742	4.75	0.007
14	Myomegalin phosphodiesterase 4D interacting protein	gi|114558492	Q5TB27	MALDI-TOF	72	14	165,585	5.36	0.011
15	major vault protein similar to lung resistance-related protein homologue	gi|19913410	Q14764	MALDI-TOF/TOF	71	29	99,551	5.34	0.008
16	Heat shock protein HSP 90-alpha 2	gi|61656603	Q14568	LC-MSMS	229	7	98,052	5.09	0.034
17	heat-shock protein hsp86	gi|194033	P07901	LC-MSMS	151	19	35,652	4.56	0.034
18	heat shock protein 70.2	gi|47523308	Q6S4N2	MALDI-TOF	73	26	70,340	5.6	0.013
19	phosphoglucomutase 5	gi|156121315	A1L598	MALDI-TOF	72	26	62,708	6.77	0.007
20	Dystrobrevin alpha (DTNA)	gi|13626507	Q9Y4J8	MALDI-TOF	61	23	84,679	6.37	0.02
21	cytoskeletal beta actin	gi|45269029		LC-MSMS	251	19	44,763	5.55	0.79
22	spermine synthase (SPSY)	gi|78369282	P52788	LC-MSMS	171	15	41,198	4.96	0.51
23	galactokinase I	gi|3603423	P51570	LC-MSMS	54	3	42246	6.04	0.014
24	galactokinase 1	gi|3603423	P51570	LC-MSMS	54	3	42,246	6.04	0.014
25	transferase, HG -phosphoribosyl	gi|224052		LC-MSMS	107	11	24,362	6.24	0.015
26	glutathione S-transferase, mu 2	gi|116047849	P08010	LC-MSMS	171	19	22,353	5.52	0.038
27	glutathione-S-transferase, mu 5	gi|25282395		LC-MSMS	62	7	22353	5.52	0.019
28	Peroxiredoxin-2 (Thioredoxin peroxidase 1)	gi|1717797	P52552	LC-MSMS	156	34	14,158	4.7	0.003

### Classification of proteins identified in the GV- and MII- stage oocytes

The 28 proteins that were either up- and down-regulated during oocyte IVM were classified according to related biological processes and molecular functions using information from the

**Figure 4:**
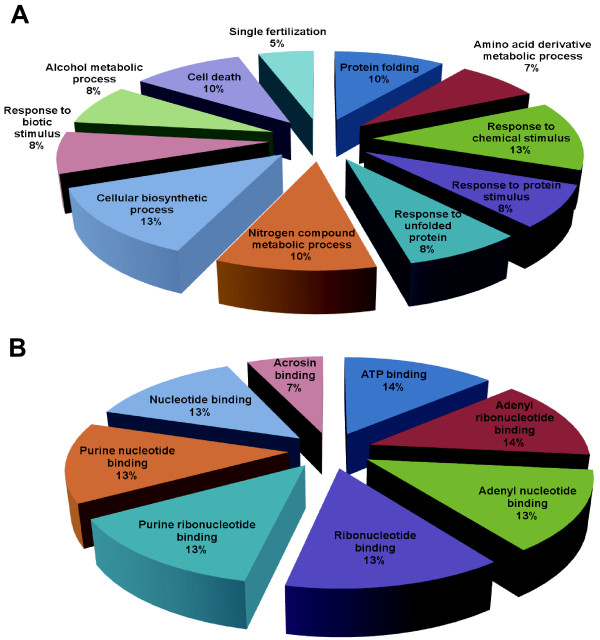
**Classification of differentially regulated proteins**. An ontological classification of differentially regulated proteins in terms of (A) biological process and (B) molecular function using the Gene Ontology http://www.geneontology.org and UniProt http://www.expasy.uniprot.org websites. The compositions of the identified proteins are presented as percentages of all individually identified proteins.

Gene Ontology http://www.geneontology.org and UniProt http://www.uniprot.org websites (Figure 4).

These differentially expressed proteins have different roles in a variety of biological processes, including protein folding (10%), the amino acid derivative metabolic process (7%), the response to chemical stimulus (13%), the response to protein stimulus (8%), the response to unfolded proteins (8%), the nitrogen compound metabolic process (10%), the cellular biosynthetic process (13%), the response to biotic stimulus (8%), the alcohol metabolic process (8%), cell death (10%) and single fertilization (5%).

Based on their molecular functions, these proteins were classified as ATP binding (14%), adenyl ribonucleotide binding (14%), adenyl nucleotide binding (13%), ribonucleotide binding (13%), purine ribonucleotide binding (13%), purine nucleotide binding (13%), nucleotide binding (13%) and acrosin binding (7%).

**Figure 5:**
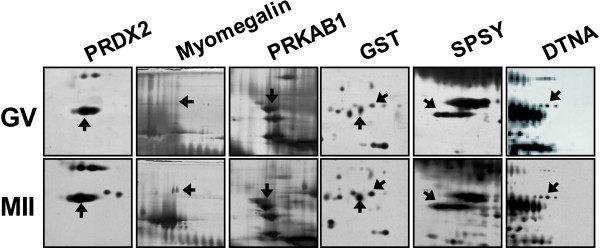
**Enlarged images of protein spots differentially expressed between the GV and M II stages**. (A) The PRDX2, myomegalin, PRKAB1, GST, SPSY and DTNA proteins were up-regulated in the M II stage when compared with the GV stage. (B) Quantitative analysis of the expression levels of these proteins. The numbered spots were identified by mass spectrometry, and the numbers correspond to their respective proteins in Tables 1 and 2.

Among these 28 proteins, we focused on functionally clustered proteins involved in redox regulation, single fertilization, cellular biosynthetic processes, ATP binding and the cAMP dependent signaling pathway, which includes the proteins PRDX 2, GST, SPSY, myomegalin, PRKAB 1, and DTNA. The corresponding protein spots for these proteins were visualized separately (Figure 5).

### Validation of differentially expressed proteins by western blot analysis in GV- and MII- stage oocytes

**Figure 6:**
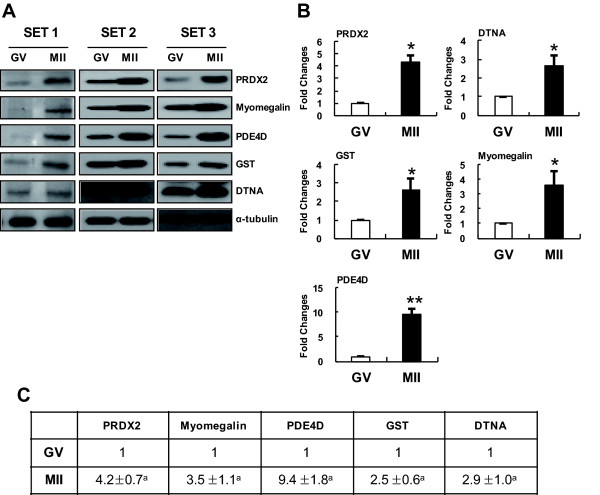
**Western-blot analyses confirming the upregulation of particular proteins during the M II stage**. (A) Western blot analyses of total protein extracts from GV and M II oocytes (lane 1, GV oocytes; lane 2, M II oocytes) using specific antibodies against proteins (PRDX2, Myomegalin, PDF4D, GST and DTNA) identified by 2-DE. Alpha-tubulin was used as a loading control. (B) Quantification of PRDX2, Myomegalin, PDF4D, GST and DTNA expression in GV and MII embryos. PRDX2, Myomegalin, PDF4D, GST and DTNA protein expression increased following MII embryos. *Value significantly differs from the control (*P <*0.05 *and **P <*0.01*). The results, which were analyzed by the TINA program, are presented in a bar graph.

For the validation of the expression differences by western blot, focus was placed on MII-stage specific up-regulated proteins that are functionally involved in redox regulation and the cAMP dependent signaling pathway. Immunoblotting with antibodies specific to PRDX 2, GST, myomegalin and DTNA was used to examine the protein expression patterns between the GV and MII stages (Figure 6). PED4D was also included in this analysis because it is a binding partner of myomegalin.

These five proteins that were identified as up-regulated proteins by 2-DE analysis were also shown to be up-regulated in the MII stage when compared with GV stage oocytes. The relative expression levels of PRDX 2, myomegalin, PDE4D, GST and DTNA were approximately 1.8-, 3.8-, 1.9-, 2.0-, 1.8- and 1.4-fold higher based on western blot analyses, respectively.

## Discussion

Oocyte IVM is influenced by many factors, such as the quality of the oocyte undergoing maturation, medium composition used for oocyte culture during in the maturation process. Although methods for quality control and for optimizing medium composition for oocyte IVM have been well known, the regulatory factors governing oocyte maturation have rarely been studied. Elucidation of the molecular networks and the mechanisms underlying the IVM process as well as identifying the regulatory factors of this process is one of the major challenges for understanding IVM and improving IVM efficacy.

There are several recent studies utilizing proteomic approaches to the study of ooctyes in other species, including the exploration of the xenopus and mouse oocyte proteomes [10-13]. These studies had been tried to find out regulatory factors during oocyte maturation and described the potential role or different molecular expression profile of specific factors in oocyte maturation. For example, Calvert et al. identified 8 highly abundant heat shock proteins (HSPs) and related chaperones in the mature mouse egg by 2-DE analysis. Vitale et al. used 2DE and mass spectrometry (MS) to identify 12 proteins that appeared to be differentially expressed between germinal vesicle (GV) and metaphase II (MII) murine oocytes [11,12]. Here, we utilized a proteomics approach using porcine oocytes maturing in vitro after their release from follicles to investigate the IVM process and define key regulatory factors.

First, we determined that this porcine model was suitable for proteomic analyses given the relatively high availability of oocytes. Pig oocytes at the GV stage could be obtained from the ovaries of slaughtered noncycling gilts through the aspiration of antral follicles of approximately 3-5 mm. To confirm this potential method, the mature oocyte ratio during IVM was measured. At the initiation of in vitro culturing of oocytes, over 70% of the oocytes were resting in the GV stage, and after 44 hr, over 80% of the oocytes cultured in vitro had reached the MII stage (Figure 1). Based on these results, we confirmed that the oocytes obtained before and after the IVM process were both suitable for proteomic analyses.

As a result (Figure 2), a total of 50 differentially expressed proteins were observed by 2-DE analysis of GV- and MII-stage oocytes, and selected spots were submitted for identification by mass spectrometry. Interestingly, some of the identified proteins, including PRDX 2, GST, SPSY, myomegalin, PDE4D, PRKAB1 and DTNA, were involved in redox regulation and the cAMP dependent signaling pathway, both of which have been correlated with oocyte maturation (Table 2, Figure 5 and 6). It has been reported that the peroxiredoxin and GST proteins, which are involved in intracellular redox regulation and protection against oxidative stress, were among the most highly abundant oocyte proteins [14]. Peroxiredoxins (PRDXs) are thioredoxin-dependent peroxide reductases localized either in the cytoplasm (PRDX1 and PRDX2) or the mitochondria (PRDX3). They represent a new type of defense system against reactive oxygen species, and their peroxidase activity relies on thioredoxin[15,16]. In addition, peroxiredoxin enzymes might participate in signaling cascades. GST exists in multiple forms, and the Mu-class has been identified in mouse spermatogenic cells, where it likely plays a role in antioxidative protection. Furthermore, the GST Mu2 and Mu5 proteins are also expressed at relatively high levels in porcine oocytes[17,18]. Taken together, these findings indicate the maintenance of high levels of antioxidant enzymes and that these enzymes play an important role in porcine oocytes during the final stages of maturation. In addition, subcellular targeting of the components of the secondary messenger cAMP-dependent pathway is thought to be essential for the intracellular signaling that leads to oocyte maturation[19]. The exact mechanism by which cAMP maintains the oocyte in meiotic arrest is, however, not fully understood. In most species, a high intracellular level of cAMP in the oocyte maintains meiotic arrest, whereas a low level of cAMP allows meiotic resumption [7,20].

Another differentially expressed protein was myomegalin, which interacts with the cyclic nucleotide phosphodiesterase PDE4D (cAMP-specific 3',5'-cyclic phosphodiesterase 4D). The up-regulated expression of myomegalin and PDE4D was verified by western blot (Figure 5 and 6). Myomegalin is known to be an anchor that helps localize components of the cAMP-dependent pathway to the Golgi/centrosomal region of the cell. In addition, cyclic nucleotide phosphodiesterases (PDEs), which are the enzymes that degrade and inactivate cAMP through the hydrolysis of cyclic nucleotides (i.e., cAMP to 5'-AMP), may play important roles in signaling compartmentalization by controlling cAMP diffusion and its ability to reach the PKA isoenzymes anchored to different organelles [21-23]. Until recently, adenosine monophosphate (AMP) was considered to be an inactive product of cAMP degradation by PDEs.

AMP, however, is now considered to be a potent allosteric activator of adenosine monophosphate kinase (PRKA; formerly known as AMPK). This member of the PRKA/SNF1 protein kinase family is a well-conserved heterotrimeric protein with a 63-kDa catalytic α subunit (PRKAA) and regulatory β (PRKAB) and γ subunits (38 kDa and 35 kDa, respectively). PRKA is an important energy-sensing enzyme that monitors cellular energy status and has been implicated as metabolic switch in an increasing number of physiological functions, including exercise, glucose uptake, glycolysis, transcriptional regulation, lipolysis, fatty acid oxidation, and sterol synthesis. PRKA also participates in the nuclear maturation of porcine oocytes, and its activation results in meiotic resumption of mouse oocytes maintained in meiotic arrest by a cAMP analog[24,25]. Furthermore, protein kinase A (PKA) belongs to a family of cAMP-dependent protein kinase that has activity that is dependent on the levels of cAMP. PKA also has several other functions in the cell, including the regulation of glycogen and sugar as well as lipid metabolism[24]. Another of the identified proteins, DTNA (dystrobrevins-alpha), interacts with PKA regulatory subunits. The direct relationship between DTNA and PKA is still unknown, but they are both cAMP-dependent, which may provide a clue to better understand the effects of their direct relationship on oocyte maturation [26,27].

## Conclusion

In conclusion, our study shed light on the mechanism responsible for the IVM process and oocytes maturation as well as identified some of the potentially key regulatory proteins involved in this molecular mechanism through the generation of proteomic profiles of porcine oocytes at different developmental stages. The information produced by this study may be beneficial not only for basic science with respect to the improvement of oocyte culture conditions, which are still far from optimal, but also for mammalian reproductive biotechnology.

## Materials and methods

### Culture Media

All chemicals used in this study were purchased from Sigma-Aldrich Corporation (St. Louis, Mo, USA) unless otherwise stated. Oocytes were cultured in tissue culture medium 199 (TCM-199; GIBCO) supplemented with 10% (v/v) porcine follicular fluid (pFF), 0.6 mM cysteine, 10 IU/mL human chorionic gonadotropin (hCG), 10 ng/ml epidermal growth factor (EGF), 5 μg/ml LH and 0.5 μg/ml follicle stimulating hormone (FSH).

### Porcine Oocyte Collection and in vitro Maturation

Porcine ovaries were collected at a local slaughterhouse and kept in a saline solution (NaCl 0.9% w/v, penicillin 100,000 IU/L, streptomycin 100 mg/L and amphotericin B 250 μg/L) at 25 to 30°C. Cumulus-oocyte complexes (COCs) were aspirated from 2 to 6 mm follicles with an 18-gauge needle and a 10-mL syringe. COCs with uniform ooplasm and a compact cumulus cell mass were placed in HEPES-buffered TALP medium containing 0.1 (v/v) polyvinyl alcohols (H-TL-PVA). The collected oocytes were washed three times with a maturation medium. Groups of 50 oocytes were placed in 500 μl of TCM-199, preincubated in 4-well multidishes (NUNC, Roskilde, Denmark) and overlaid with 500 μl mineral oil in a humidified atmosphere at 38.4°C and 5% CO_2_. The oocytes were then incubated for the first 20~22 h with 10% (v/v) pFF, 0.6 mM cysteine, 10 IU/mL hCG, 10 ng/ml EGF, 5 μg/ml LH and 0.5 μg/ml FSH, washed three times in maturation medium and returned for another 20~22-h incubation in 500 μl of maturation medium without hormones. For the collection of GV-stage oocytes, the oocytes were denuded immediately after selection by vortexing for 7 min at medium speed in 1 ml of 0.01% hyaluronidase. For the collection of metaphase II stage oocytes, the oocytes were denuded by vortexing for 3 min in 0.01% hyaluronidase. The cumulus-free GV- and MII-stage oocytes were then washed three times in PBS and frozen immediately in a minimal volume of PBS at -80°C.

Morphological evaluations of the oocytes were performed to verify that they were either at stage GV or MII and to determine the quality of the oocytes that would be used in two-dimensional gel electrophoresis (2-DE).

The oocytes were mounted on microscope slides with vaseline, covered with a glass coverslip, and fixed in a 3:1 ethanol:acetic acid mixture for 24 hr. The cells were stained with 5% orcein in a solution of 50% aqueous acetic acid and 1% sodium citrate. After destaining in 40% acetic acid, the slides were examined with a microscope. The criterion for inclusion in the proteomics study was that at least 90% of the oocytes from a single collection had reached the appropriate maturation stage.

### Proteomic analysis

Total protein extracts were prepared from porcine oocytes using a protein extraction solution (1.0 mM PMSF, 1.0 mM EDTA, 1 M pepstatin A, 1 M leupeptin, and 0.1 M aprotinin). 2-DE analysis was performed using an IPGphor IEF unit as described previously[28]. The silver-stained gels were scanned with an ImageScanner (Amersham, USA) and analyzed with Phoretix Expression software (ver. 2005; Nonlinear Dynamics, UK). Destaining and in-gel tryptic digestion of the protein spots were performed as described[29]. Briefly, Xcise (Shimadzu Biotech Co., Japan), an automatic sample preparation system, was used for in-gel digestion, desalting, and plating onto a MALDI-TOF plate. Desalting was performed with ZipTipC^18 ^(Millipore, Bedford, MA, USA), and plating was performed using a 4-hydroxy-α-cyano-cinnamic acid (HCCA) matrix solution. The in-gel-digested peptides were analyzed using an ultraflex-TOF/TOF (Bruker Daltonics, Germany) mass spectrometer. The mass spectra were calibrated and processed using the Flex Analysis and BioTool 2.2 software (Bruker Daltonics, Germany). Peptide mass fingerprinting (PMF) ion searches were performed using the Mascot 2.1 software http://www.matrixscience.com integrated with BioTool 2.2. The MSDB (version 20060831: 3239079 sequences) and NCBInr (version 20080125: 5872070 sequences) protein databases were searched using the following Mascot settings: taxonomy, Homo sapiens; one incomplete tryptic cleavage allowed; peptide tolerance, 50-100 ppm; fragment tolerance, 0.5 Da; monoisotopic mass; 1+ peptide charge state with HCCA protonation, alkylation of cysteine by carbamidomethylation as a fixed modification, and oxidation of methionine as a variable modification. For each search, statistically significant (p < 0.05) matches were regarded as correct hits. The threshold score for the MSDB was 67, and the threshold score for the NCBI database was 67-78. Two criteria were used for further analysis: matches to porcine proteins were selected over matches to proteins from other species even if the non-porcine proteins had higher ranked hits, and proteins with theoretical pIs and MWs that matched those of the corresponding protein in the 2-DE gel were selected preferentially, even if other proteins had higher ranked hits.

### MALDI-TOF calibration

The peptides used for the MALDI-TOF calibration were bradykinin (1-7)_(M+H)+_mono (757.399), angiotensin_ll_(M+H)+_mono (1046.541), angiotensin_1_(M+H)+_mono (1296.684), substance_P_(M+H)+_mono (1347.735), bombesin_(M+H)+_mono (1619.822), renin_substrate_(M+H)+_mono (1758.93), ACTH_clip(1-17)(M+H)+_mono (2093.086), ACTH_clip(18-39)(M+H)+_mono (2465.198), and somatostatin(28) (M+H)+_mono (3147.471).

### LC-MS/MS analysis

The tryptic peptides were extracted three times to ensure recovery of all the peptides from the gel particles. The recovered peptides were concentrated by drying the final volume of the extracts in a vacuum centrifuge. To prepare the samples for liquid chromatography tandem mass spectrometry (LC-MS/MS) analysis, the concentrated peptides were mixed with 20 μl of 0.1% formic acid in 3% acetonitrile. Nano LC of the tryptic peptides was performed using a Waters Nano LC system equipped with a Waters C18 nano column (75 μm × 15 cm nanoAcquity™ UPLCTM columns). Binary solvent A1 contained 0.1% formic acid in water, and binary solvent B1 contained 0.1% formic acid in acetonitrile. Samples (5 μl) were loaded onto the column, and the peptides were eluted from the column with a gradient ranging from 2% to 40% binary solvent B1 for 30 min at a rate of 0.4 μl/min. The lock mass, [Glu1]fibrin peptide at 400 fmol/μl, was delivered from the auxiliary pump of the Nano LC system at a rate of 0.3 μl/min to the reference sprayer of the NanoLockSpray™ source.

### Mass spectrometer configuration

Mass spectrometry analysis of the tryptic peptides was performed using a Waters Synapt™HDMS. The mass spectrometer was operated in V-mode for all measurements, and all analyses were performed using a positive-mode Nano ESI with a NanoSpray source. The lock mass channel was sampled every 30 s. The mass spectrometer was calibrated using a [Glu^1^] fibrinopeptide solution delivered at a rate of 400 fmol/μl through the reference sprayer of the NanoLockSpray source. Accurate mass LC-MS/MS data were collected using the data-dependent acquisition mode.

### Data processing and protein identification

A continuum of LC-MS/MS data was processed, and the data were automatically smoothed, background-subtracted, centered and deisotoped. In addition, the charge state was reduced, and the masses were corrected based on the reference scans. Ion detection, clustering and normalization were performed using PLGS. The processed data were used to search the IPI human database using the Protein Lynx Global Server (PLGS), version 2.3 (Waters)[30,31]. Processed ions were sequenced and mapped against the IPI human database using the PLGS and MASCOT DAEMON programs http://www.matrixscience.com. The PLGS search parameters were defined by the software using the system settings. Peptides were restricted to trypsin fragments with a maximum of one missed cleavage and cysteine carbamidomethylation.

### Western blot analysis

Aliquots of 30 μg of protein extracts from EBs(embryoid body) and beating and non-beating cells were separated by SDS-PAGE. The proteins were then transferred to a nitrocellulose membrane, which was blocked for 2 h at 25°C with 3% wt/vol BSA/TBST (10 mM Tris HCl, pH 7.4, 140 mM NaCl, 0.1% Tween-20) and then incubated with the polyclonal antibodies for PRDX2 (1:1000, Santa Cruz Biotechnology), myomegalin (1:1000, Santa Cruz Biotechnology), PDE4D (1:1000, Chemicon), GST (1:1000, Santa Cruz Biotechnology), DTNA (1:1000, Santa Cruz Biotechnology), and α-tubulin (1:3000, Santa Cruz Biotechnology) at 4°C overnight. After washing with TBST, the membranes were incubated with the appropriate secondary antibodies for 1 h at 37°C and visualized by enhanced chemiluminescence (Amersham Biosciences). After the membranes were scanned, the signal intensity of each band was determined using LAS 3000 (Fuji Photo Film Co., Ltd). The relative protein levels in each sample were normalized against the level of α-tubulin.

### Statistical analysis

All quantitative data are expressed as means ± SD of three independent experiments. Statistical significance between groups were evaluated via one-way analysis of variance (ANOVA), followed by paired two-tailed Student's *t*-test.

## Competing interests

The authors declare that they have no competing interests.

## Authors' contributions

J and JS carried out the conception and design, data analysis and interpretation, and manuscript writing. YJ, DW and TH performed western blotting and statistical analysis. Y, HK, NJ, SB, KS and HM participated in its design and coordination and help to collection samples. DS and JI participated in conception and design, data analysis and interpretation, financial support and administrative support. All authors read and approved the final manuscript.

## References

[B1] Kikuchi K Somfai T Nakai M Nagai T Appearance, fate and utilization of abnormal porcine embryos produced by in vitro maturation and fertilization Soc Reprod Fertil Suppl 2009 66 135 147 19848277

[B2] Moor R Dai Y Maturation of pig oocytes in vivo and in vitro Reprod Suppl 2001 58 91 104 11980205

[B3] Novak SPF Savard C Tremblay K Sirard MA Identification of porcine oocyte proteins that are associated with somatic cell nuclei after co-incubation Biol Reprod 2004 71 1279 1289 10.1095/biolreprod.103.027037 15201196

[B4] Ellederova Z Halada P Man P Kubelka M Motlik J Kovarova H Protein patterns of pig oocytes during in vitro maturation Biol Reprod 2004 71 1533 1539 10.1095/biolreprod.104.030304 15229143

[B5] Fair T Carter F Park S Evans AC Lonergan P Global gene expression analysis during bovine oocyte in vitro maturation Theriogenology 2007 68 Suppl 1 S91 97 17512044 10.1016/j.theriogenology.2007.04.018

[B6] Susor A Ellederova Z Jelinkova L Halada P Kavan D Kubelka M Kovarova H Proteomic analysis of porcine oocytes during in vitro maturation reveals essential role for the ubiquitin C-terminal hydrolase-L1 Reproduction 2007 134 559 568 10.1530/REP-07-0079 17890291

[B7] Liang CGHL Zhong ZS Chen DY Schatten H Sun QY Cyclic adenosine 3',5'-monophosphate-dependent activation of mitogen-activated protein kinase in cumulus cells is essential for germinal vesicle breakdown of porcine cumulus-enclosed oocytes Endocrinology 2005 146 4437 4444 10.1210/en.2005-0309 16002524

[B8] Ozawa MNT Somfai T Nakai M Maedomari N Fahrudin M Karja NW Kaneko H Noguchi J Ohnuma K Yoshimi N Miyazaki H Kikuchi K Comparison between effects of 3-isobutyl-1-methylxanthine and FSH on gap junctional communication, LH-receptor expression, and meiotic maturation of cumulus-oocyte complexes in pigs Mol Reprod Dev 2008 75 857 866 10.1002/mrd.20820 18022826

[B9] Prather RS Ross JW Isom SC Green JA Transcriptional, post-transcriptional and epigenetic control of porcine oocyte maturation and embryogenesis Soc Reprod Fertil Suppl 2009 66 165 176 19848279 PMC2842954

[B10] Marteil G D'Inca R Pascal A Guitton N Midtun T Goksoyr A Richard-Parpaillon L Kubiak JZ EP45 accumulates in growing Xenopus laevis oocytes and has oocyte-maturation-enhancing activity involved in oocyte quality J Cell Sci 123 1805 1813 10.1242/jcs.063305 20427318

[B11] Ma M Guo X Wang F Zhao C Liu Z Shi Z Wang Y Zhang P Zhang K Wang N Protein expression profile of the mouse metaphase-II oocyte J Proteome Res 2008 7 4821 4830 10.1021/pr800392s 18803416

[B12] Zhang P Ni X Guo Y Guo X Wang Y Zhou Z Huo R Sha J Proteomic-based identification of maternal proteins in mature mouse oocytes BMC Genomics 2009 10 348 10.1186/1471-2164-10-348 19646285 PMC2730056

[B13] D'Inca R Marteil G Bazile F Pascal A Guitton N Lavigne R Richard-Parpaillon L Kubiak JZ Proteomic screen for potential regulators of M-phase entry and quality of meiotic resumption in Xenopus laevis oocytes J Proteomics 73 1542 1550 10.1016/j.jprot.2010.03.017 20394845

[B14] Berendt FJ Frohlich T Bolbrinker P Boelhauve M Gungor T Habermann FA Wolf E Arnold GJ Highly sensitive saturation labeling reveals changes in abundance of cell cycle-associated proteins and redox enzyme variants during oocyte maturation in vitro Proteomics 2009 9 550 564 10.1002/pmic.200700417 19137544

[B15] Wang S Huang W Shi H Lin C Xie M Wang J Localization and expression of peroxiredoxin II in the mouse ovary, oviduct, uterus, and preimplantation embryo Anat Rec (Hoboken) 293 291 297 19899112 10.1002/ar.21031

[B16] Leyens G Knoops B Donnay I Expression of peroxiredoxins in bovine oocytes and embryos produced in vitro Mol Reprod Dev 2004 69 243 251 10.1002/mrd.20145 15349835

[B17] Raza HRM Fang JK Avadhani NG Multiple isoforms of mitochondrial glutathione S-transferases and their differential induction under oxidative stress Biochem J 2002 366 45 55 12020353 10.1042/BJ20020533 PMC1222767

[B18] Rabahi FBS Sirois J Beckers JF Silversides DW Lussier JG High expression of bovine alpha glutathione S-transferase (GSTA1, GSTA2) subunits is mainly associated with steroidogenically active cells and regulated by gonadotropins in bovine ovarian follicles Endocrinology 1999 140 3507 3517 10.1210/en.140.8.3507 10433206

[B19] Kim JS Cho YS Song BS Wee G Park JS Choo YK Yu K Lee KK Han YM Koo DB Exogenous dibutyryl cAMP affects meiotic maturation via protein kinase A activation; it stimulates further embryonic development including blastocyst quality in pigs Theriogenology 2008 69 290 301 10.1016/j.theriogenology.2007.09.024 17977589

[B20] Mattioli M Galeati G Barboni B Seren E Concentration of cyclic AMP during the maturation of pig oocytes in vivo and in vitro J Reprod Fertil 1994 100 403 409 10.1530/jrf.0.1000403 8021856

[B21] Bolger GB Peden AH Steele MR MacKenzie C McEwan DG Wallace DA Huston E Baillie GS Houslay MD Attenuation of the activity of the cAMP-specific phosphodiesterase PDE4A5 by interaction with the immunophilin XAP2 J Biol Chem 2003 278 33351 33363 10.1074/jbc.M303269200 12810716

[B22] Fleming YM Frame MC Houslay MD PDE4-regulated cAMP degradation controls the assembly of integrin-dependent actin adhesion structures and REF52 cell migration J Cell Sci 2004 117 2377 2388 10.1242/jcs.01096 15126637

[B23] Verde IPG Salanova M Zhang G Wang S Coletti D Onuffer J Jin SL Conti M Myomegalin is a novel protein of the golgi/centrosome that interacts with a cyclic nucleotide phosphodiesterase J Biol Chem 2001 276 11189 11198 10.1074/jbc.M006546200 11134006

[B24] Skalhegg BS Tasken K Specificity in the cAMP/PKA signaling pathway. Differential expression, regulation, and subcellular localization of subunits of PKA Front Biosci 2000 5 D678 693 10.2741/Skalhegg 10922298

[B25] Mayes MA Laforest MF Guillemette C Gilchrist RB Richard FJ Adenosine 5'-monophosphate kinase-activated protein kinase (PRKA) activators delay meiotic resumption in porcine oocytes Biol Reprod 2007 76 589 597 10.1095/biolreprod.106.057828 17167165

[B26] Lien CF Vlachouli C Blake DJ Simons JP Gorecki DC Differential spatio-temporal expression of alpha-dystrobrevin-1 during mouse development Gene Expr Patterns 2004 4 583 593 10.1016/j.modgep.2004.01.015 15261837

[B27] Ceccarini M Grasso M Veroni C Gambara G Artegiani B Macchia G Ramoni C Torreri P Mallozzi C Petrucci TC Macioce P Association of dystrobrevin and regulatory subunit of protein kinase A: a new role for dystrobrevin as a scaffold for signaling proteins J Mol Biol 2007 371 1174 1187 10.1016/j.jmb.2007.06.019 17610895

[B28] Lee KA Shim JH Kho CW Park SG Park BC Kim JW Lim JS Choe YK Paik SG Yoon DY Protein profiling and identification of modulators regulated by the E7 oncogene in the C33A cell line by proteomics and genomics Proteomics 2004 4 839 848 10.1002/pmic.200300626 14997504

[B29] O'Neill EE Brock CJ von Kriegsheim AF Pearce AC Dwek RA Watson SP Hebestreit HF Towards complete analysis of the platelet proteome Proteomics 2002 2 288 305 10.1002/1615-9861(200203)2:3<288::AID-PROT288>3.0.CO;2-0 11921445

[B30] Wang H Qian WJ Mottaz HM Clauss TR Anderson DJ Moore RJ Camp DG Khan AH Sforza DM Pallavicini M Development and evaluation of a micro- and nanoscale proteomic sample preparation method J Proteome Res 2005 4 2397 2403 10.1021/pr050160f 16335993 PMC1781925

[B31] Liu T Qian WJ Strittmatter EF Camp DG Anderson GA Thrall BD Smith RD High-throughput comparative proteome analysis using a quantitative cysteinyl-peptide enrichment technology Anal Chem 2004 76 5345 5353 10.1021/ac049485q 15362891

